# Refractory Atrial Fibrillation With Rapid Ventricular Rate in a Patient With Small Cell Carcinoma of the Lung Encasing the Right Pulmonary Artery: A Case Report and Insight Into Therapeutic Options

**DOI:** 10.7759/cureus.16027

**Published:** 2021-06-29

**Authors:** Kelechi E Emmanuel, Nichole Jensen, Uche Anyanwagu

**Affiliations:** 1 Internal Medicine, University of Pittsburgh Medical Center Pinnacle, Harrisburg, USA; 2 Family Medicine, University of Nottingham, Royal Derby Hospital, Nottingham, GBR

**Keywords:** atrial fibrillation, chemotherapy, small cell lung cancer, syndrome of inappropriate anti diuretic hormone, anti-arrhythmia

## Abstract

Atrial fibrillation is the most common sustained cardiac arrhythmia. While there have been reports of atrial fibrillation caused by the compression of pulmonary veins, we have not found reports of atrial fibrillation caused by the compression of the pulmonary artery. This report highlights the possible pathophysiology and management of atrial fibrillation in a patient with small cell lung cancer.

The patient was admitted for hyponatremia secondary to syndrome of inappropriate antidiuretic hormone (SIADH) but subsequently developed tachycardia which progressed to atrial flutter and atrial fibrillation. Antiarrhythmics were ineffective until the patient received his first palliative chemotherapy for his small cell lung cancer. Subsequently, rate control was achieved with sotalol, with eventual conversion back to sinus rhythm.

Management of atrial fibrillation is complex and sometimes depends on the underlying etiology. Early chemotherapy, in addition to antiarrhythmic drugs, may be beneficial in the management of patients with small cell lung cancer and atrial fibrillation. The CHA2DS2-VASc score does not take active malignancy into account and anti-coagulation should be evaluated on a case-by-case basis in this patient population.

## Introduction

Atrial fibrillation and atrial flutter are very common in the general population. With a prevalence of 0.12%-0.16% in people younger than 49 years, 3.7%-4.2% in persons older than 60 years, and 10%-17% in those older than 80 years, atrial fibrillation is the most common sustained cardiac arrhythmia in the Western world [1]. In the United States alone, it is estimated that at least three million to six million people have atrial fibrillation [2]. It is commonly associated with different cancers, including metastatic cancers. Cross-sectional studies show that atrial fibrillation is more prevalent in patients with cancer when compared to their age-matched counterparts without cancer (3.6% in patients with cancer versus 1.5% in controls who had no diagnosis of cancer) [3]. In a study done with 159,615 patients with lung cancer, the prevalence of atrial fibrillation was found to be 6.29% [4]. Atrial fibrillation is often triggered by different tumors, including intra-cardiac tumors [5], and is known to contribute to overall morbidity in cancer patients. The effect of atrial fibrillation on mortality in lung cancer patients is increasingly recognized. It has been reported that new-onset atrial fibrillation is independently associated with a higher incidence of heart failure. In addition, patients with lung cancer and atrial fibrillation have also been shown to have an increased risk of one-year mortality [6]. In spite of this, there is a paucity of data in the overall pathophysiology and management of atrial fibrillation in patients with small-cell lung cancer. Previous cases have reported atrial fibrillation in patients with lung cancer compressing the left pulmonary vein and left atrium but we found no reports of atrial fibrillation secondary to right pulmonary artery compression.

We present a case of atrial flutter and atrial fibrillation in a 49-year-old male, caused by small cell lung cancer compressing the right pulmonary artery.

## Case presentation

A previously healthy 49-year-old male presented to the ED for evaluation of hyponatremia (sodium 122 mmol/L) which was incidentally discovered during an outpatient laboratory workup for new-onset unexplained recurrent syncopal episodes, dyspnea with exertion, and a 20-pound unintentional weight loss. He was not on any regular medications, but he had a 37-pack-year smoking history. The patient had initially gone to his primary care physician with the previously mentioned complaints. At that time, his vitals were stable; laboratory investigations were done and the remarkable result was the sodium of 122 mmol/L for which he was sent to the ER for further evaluation. 

**Figure 1:**
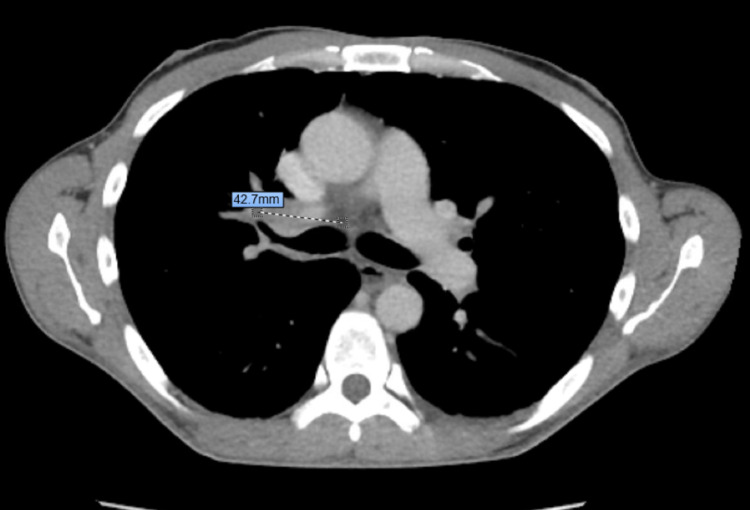
CT of the chest showing a mass (dotted line) compressing the right pulmonary artery and the right main bronchus.

On presentation to the ER, he was in no acute distress. His tympanic temperature was 37 degrees Celsius, heart rate 78 beats per minute, and blood pressure 110/70 mmHg. His lung examination showed decreased breath sounds bilaterally at the bases, and the rest of his physical examination was unremarkable. His EKG showed normal sinus rhythm, the chest X-ray was remarkable for possible pneumonitis of the right middle lobe with hilar adenopathy, while CT of the chest showed an ill-defined infiltrative mass in the right hilum, associated with narrowing of the right pulmonary arteries and occlusion of the right middle lobe bronchi with complete middle lobe atelectasis secondary to endobronchial obstruction (Figure 1). CT abdomen and pelvis were also done on admission which showed liver masses that were suspected to represent metastatic lesions from small cell lung cancer.

**Figure 2:**
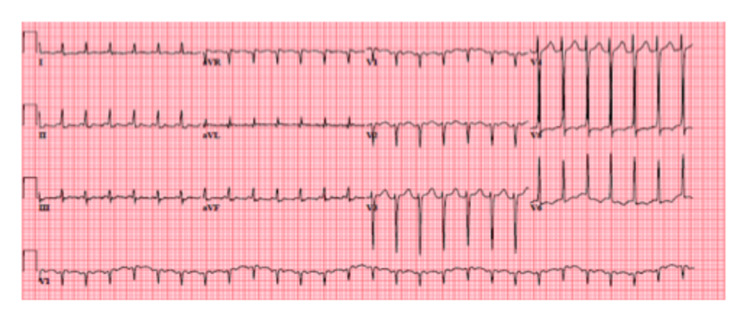
ECG showing atrial flutter.

**Figure 3:**
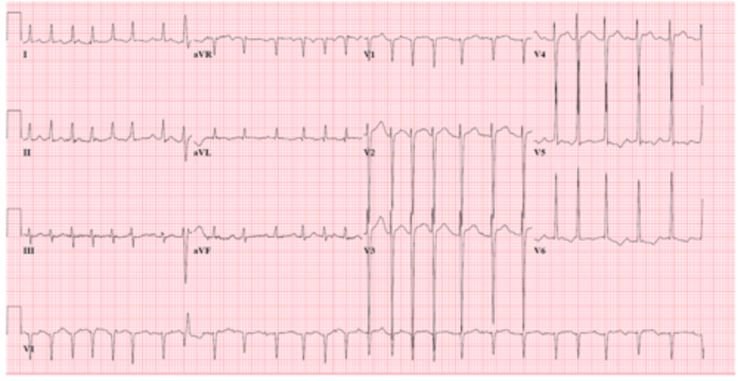
ECG showing atrial fibrillation with rapid ventricular rate.

The results of the laboratory investigations done in the ER showed sodium 122 mmol/L, potassium 3.8 mmol/L, serum osmolality was 270 mOsm/kg, and urine osmolality was 32 mEq/L. He was diagnosed with hyponatremia secondary to syndrome of inappropriate antidiuretic hormone (SIADH) and was admitted to the general inpatient unit for correction of his hyponatremia and further evaluation of his newly diagnosed lung mass. On day seven of his admission, he developed intermittent palpitations. Telemetry revealed sinus rhythm with paroxysms of frequent premature atrial complexes strung together into runs of atrial flutter. A new EKG was obtained which showed atrial flutter (Figure 2). His atrial flutter persisted and progressed over the course of days to persistent atrial fibrillation (Figure 3).

His new-onset atrial fibrillation was investigated. His laboratory results revealed normal thyroid-stimulating hormone, electrolytes, and lipid levels. There was no known or newly diagnosed structural heart disease. Given that there was no cause found for his arrhythmia, it was attributed to be a complication of his small cell lung cancer.

Because the patient was hemodynamically stable, he was medically managed for the atrial fibrillation with scheduled intravenous diltiazem and metoprolol pushes. His rhythm did not convert back to normal sinus rhythm, but his heart rate was briefly controlled on these agents. His heart rate stayed between 80 beats per minute to 110 beats per minute for some days but quickly reverted to fast ventricular rates ranging from 130 beats per minute to 150 beats per minute despite the use of dual atrial-ventricular blocking agents. He was started on continuous diltiazem infusion and later transitioned to sotalol with a mild decrease in his heart rate (range was between 110 beats per minute and 125 beats per minute). The decision to place the patient on anticoagulation for stroke prophylaxis was based on the CHA2DS2-VASc score alone. His CHA2DS2-VASc score was one, so no anticoagulation was started.

The patient was evaluated by the in-house oncologist during the course of his admission and he was offered palliative intent chemotherapy for his metastatic small cell lung cancer. On day 10 of his admission, the patient received the first round of chemotherapy for his small cell lung cancer. Shortly after that, his heart rate slowed down, and he eventually converted to a normal sinus rhythm on day 14 of his admission. He was maintained on the same dose of sotalol with frequent ECG to monitor his QTc. 

Unfortunately, three months after his diagnosis, this patient passed away due to hypoxic respiratory failure, secondary to complications of his metastatic small cell lung cancer.

## Discussion

**Figure 4:**
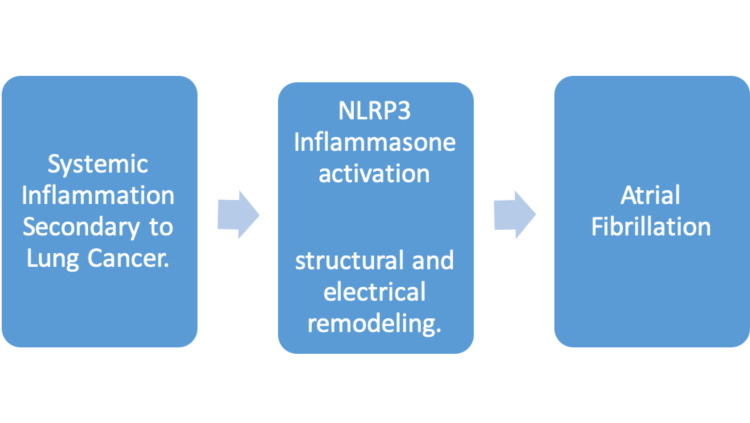
Schematic representation of the relationship between lung cancer, NLRP3 inflammasome activation, and atrial fibrillation. NLRP3: Nod-like receptor protein 3.

Though a common cardiac arrhythmia, the pathophysiology of atrial fibrillation in cancer patients is not completely understood, but systemic inflammation [7], atrial tissue compression causing altered conduction, and hyper-coagulation of malignancy that leads to the formation of pulmonary microemboli are the most accepted explanations. Other proposed pathophysiology was an enhanced cardiomyocyte inflammasome [8]. Another possible pathophysiology is an inflammation caused by cancer which leads to structural and electrical remodeling, which can, in turn, lead to atrial fibrillation [9]. External compression of the cardiac tissue by abnormal thoracic structures has also been proposed as a less common cause of atrial fibrillation, as uniquely characterized by its reversibility when the source of compression is removed [10]. The left atrium and pulmonary veins were the most reported location of involvement. This was thought to be triggered by atrial ectopic beats from muscle fibers extending from the left atrium into the pulmonary veins [11]. The involvement of the pulmonary artery (which is in close proximity to the pulmonary vein) in the reported case highlights the possible mass effect of cancer deposits as a cause/trigger of atrial fibrillation. Although atrial fibrillation secondary to compression may be underestimated given the difficulty in proving the relationship between compression and arrhythmia, other cardiac pathologies such as heart failure as a result of external compression have been easily proven due to the complete resolution of the disease with relief of the mass effect [12]. In another described pathophysiology of atrial fibrillation as a result of small cell lung cancer, Kong H et al. demonstrated that the NOD-like receptor protein 3 (NLRP) inflammasome, was upregulated in small cell lung cancer and lung adenocarcinoma [13]. This biomarker has also been studied in the pathogenesis of atrial fibrillation and it has been shown that increased levels of NLRP are present in patients with atrial fibrillation (Figure 4).

The management of atrial fibrillation in patients with lung cancer is complex and challenging irrespective of the location of the tumor. Although the tumor, in this case, was in the right pulmonary artery, the management was the same as that for tumors in other locations. Similar to the general population, antiarrhythmic drugs are often employed in the management of atrial fibrillation in patients with small cell lung cancer. The use of beta-blockers to suppress atrial fibrillation caused by pulmonary vein automaticity was reported by Mahida et al. [14]. However, in our case, we failed to gain sustained control with two atrioventricular nodal blocking agents. Rather, successful management could only be achieved with sotalol after the first dose of palliative chemotherapy.

Our finding was further corroborated by two similar reports. In one report by Amin et al., a case of atrial fibrillation was converted to and maintained in normal sinus rhythm using metoprolol after a successful tumor reduction with chemotherapy in a patient with atrial fibrillation and non-small cell lung cancer [15]. Another similar case was reported by Ahmed et al., where a patient with atrial fibrillation as a result of small cell lung cancer had an auto conversion to normal sinus rhythm after chemotherapy [16].

Our patient’s cancer progressed rapidly despite chemotherapy because he presented late in the course of his disease, with a grave prognosis from the time of diagnosis, however, after receiving chemotherapy, he maintained sinus rhythm with sotalol, a class three antiarrhythmic agent.

Anticoagulation in lung cancer patients with atrial fibrillation is challenging because cancer is both a hypercoagulable state and a condition that increases bleeding risk in patients [17]. Our patient did not receive anticoagulation; this is not always the case. In the general population, anticoagulation in patients with atrial fibrillation is based on their risk for thrombosis, which is calculated using the CHA2DS2-VASc score and less commonly, the HAS-BLED score. In cancer patients, anticoagulation is a clinical judgment based on the risk-benefit ratio.

Proposed therapeutic options

This case demonstrates that early chemotherapy portends a potentially successful strategy for the achievement and/or maintenance of normal sinus rhythm in patients with atrial fibrillation in the presence of small cell lung cancer. Although this is a proposed treatment strategy, it is important to remain cognizant of the dilemma that stems from the fact that anti-cancer therapy has been also shown to be arrhythmogenic and that drug-drug interactions have been reported between newer targeted cancer therapies and antiarrhythmic drugs and more research is needed in this area to guide the choice of therapy. Anticoagulation for the prevention of stroke in patients with atrial fibrillation and a CHA2D-VASc2 score of two or more in men and one or more in women is standard of care. This score, however, does not take into account the patients with active malignancy who are at risk of both thromboembolic as well as bleeding events [17]. Anticoagulation in this patient population should therefore be made on a case-by-case basis and more research is required in these fields.

## Conclusions

This is the first reported case of atrial flutter and subsequent atrial fibrillation triggered by a right hilar tumor compromising the right pulmonary artery. Small cell lung cancer tends to be centrally located and rapidly progressive and has the potential to involve cardiovascular structures and cause compression. Although there are limited data on the risk of developing atrial fibrillation in small cell lung cancer patients, it is hypothesized that the proportion of tumor invasion and compression, in consideration with other etiologies, drives the development of atrial fibrillation. These etiologies include tumor size and the presence of metastasis. Early palliative chemotherapy plays a significant role in reducing tumor invasion and contributes to the successful management of atrial fibrillation in this population. Therapies targeted towards blocking the NLRP inflammasome biomarker also hold promise and future study is needed to generate evidence to improve management.
